# Guided de-escalation of DAPT in acute coronary syndrome patients undergoing percutaneous coronary intervention with BVS implantation: a post-hoc analysis from the randomized TROPICAL-ACS trial

**DOI:** 10.1007/s11239-019-01811-2

**Published:** 2019-02-09

**Authors:** Lukasz Koltowski, Mariusz Tomaniak, Lisa Gross, Bartosz Rymuza, Michal Kowara, Radoslaw Parma, Anna Komosa, Mariusz Klopotowski, Claudius Jacobshagen, Tommaso Gori, Daniel Aradi, Kurt Huber, Martin Hadamitzky, Steffen Massberg, Maciej Lesiak, Krzysztof J. Filipiak, Adam Witkowski, Grzegorz Opolski, Zenon Huczek, Dirk Sibbing

**Affiliations:** 1 1st Department of Cardiology, Medical University of Warsaw, ul. Banacha 1a, 02-097 Warsaw, Poland; 2 Department of Cardiology, LMU München, Munich, Germany; 3 Division of Cardiology and Structural Heart Diseases, SMK in Katowice, Medical University of Silesia, Katowice, Poland; 4 First Department of Cardiology, Faculty of Medicine II, Poznan University of Medical Sciences, Poznan, Poland; 5 Department of Interventional Cardiology and Angiology, Institute of Cardiology, Warsaw, Poland; 6 Department of Cardiology und Pneumology, Heart Centre/Georg-August-University Göttingen, Göttingen, Germany; 7 Department of Cardiology, University Medical Center and DZHK Rheim-Main, Mainz, Germany; 8 Heart Centre Balatonfüred and Heart and Vascular Centre, Semmelweis University, Budapest, Hungary; 9 3rd Medical Department, Cardiology and Intensive Care Medicine, and Sigmund Freud Private University, Medical School, Vienna, Austria; 10 Department of Radiology, Deutsches Herzzentrum, Munich, Germany; 11 DZHK (German Centre for Cardiovascular Research), Munich Heart Alliance, Munich, Germany

**Keywords:** Bioresorbable vascular scaffold, Prasugrel, Clopidogrel, Platelet function testing

## Abstract

To investigate the safety and efficacy of an early platelet function testing (PFT)-guided de-escalation of dual antiplatelet treatment (DAPT) in acute coronary syndrome (ACS) patients undergoing percutaneous coronary intervention (PCI) with bioresorbable vascular scaffolds (BVS). Early DAPT de-escalation is a new non-inferior alternative to 12-months DAPT in patients with biomarker positive ACS treated with stent implantation. In this post-hoc analysis of the TROPICAL-ACS trial, which randomized 2610 ACS patients to a PFT-guided DAPT de-escalation (switch from prasugrel to clopidogrel) or to control group (uniform prasugrel), we compared clinical outcomes of patients (n = 151) who received a BVS during the index PCI. The frequency of the primary endpoint (cardiovascular death, myocardial infarction, stroke or BARC ≥ 2 bleeding) was 8.8% (n = 6) in the de-escalation group vs. 12.0% (n = 10) in the control group (HR 0.72, 95% CI 0.26–1.98, p = 0.52) at 12 months. One early definite stent thrombosis (ST) occurred in the control group (day 19) and 1 possible ST (sudden cardiovascular death) in the de-escalation group (day 86), both despite prasugrel treatment and in a background of high on-treatment platelet reactivity assessed at day 14 after randomization (ADP-induced platelet aggregation values of 108 U and 59 U, respectively). A PFT-guided DAPT de-escalation strategy could potentially be a safe and effective strategy in ACS patients with BVS implantation but the level of platelet inhibition may be of particular importance. This hypothesis-generating post-hoc analysis requires verification in larger studies with upcoming BVS platforms.

## Highlights

- An early PFT-guided DAPT de-escalation strategy seem to be an attractive alternative to the current DAPT regime in ACS patients treated with BVS
- A single PFT assessment performed 14 days after hospital discharge allows for a safe and effective switch from prasugrel to clopidogrel in two third of cases
- Guided DAPT de-escalation strategy could potentially decrease the bleeding risk, increase drug adherence and lower costs

## Introduction

In the randomized Testing Responsiveness To Platelet Inhibition On Chronic Antiplatelet Treatment For Acute Coronary Syndromes (TROPICAL-ACS) trial, a platelet function testing (PFT)-guided dual antiplatelet therapy (DAPT) de-escalation strategy with an early switch from prasugrel to clopidogrel was found to be equally effective and safe when compared to standard treatment with uniform and potent platelet inhibition in biomarker positive acute coronary syndrome (ACS) patients undergoing percutaneous coronary intervention (PCI) [1, 2]. Additionally, among younger ACS patients, there was a net clinical benefit from PFT-guided DAPT de-escalation, driven by a reduction in bleeding events during long-term treatment [3]. However, those observations were mainly derived from a study population treated with latest generation drug eluting stents (DES) in whom the largest benefits of potent platelet inhibition for protection against ischemic complications are observed early after PCI, while the risk of bleeding events persists during chronic antiplatelet treatment [4–6].

Follow-up data from a number of randomized controlled clinical trials as well as data from dedicated registries showed an increased ischemic risk after bioresorbable vascular scaffold (BVS) implantation, reportedly related to the specific characteristics of the device [7, 8]. These include a more demanding implantation technique and presence of quadratic thicker struts associated with larger in-scaffold areas exposed to high endothelial shear stress leading to release of potent platelet agonists (i.e. adenosine diphosphate and thromboxane A_2_) with eventually greater induction of platelet activation and increased thrombogenicity [9–11]. Late scaffold thrombosis has been associated with DAPT discontinuation [12]. A prolonged DAPT beyond 12-months has been advocated in recent 2017 ESC DAPT guidelines [13]. However, this kind of strategy does not take into account the individual response to antiplatelet treatment and may unnecessarily increase bleeding risk in adequate responders to clopidogrel. Therefore, a PFT-guided de-escalation of DAPT from a potent P2Y_12_ inhibitor to clopidogrel may offer an attractive alternative DAPT strategy especially for patients with BVS implantation and in whom sufficient and supposedly extended (> 12 months) dual platelet inhibition seems mandatory. In this respect, the recently released 2018 ESC/EACTS Guidelines on myocardial revascularization have included a PFT-guided DAPT de-escalation as a treatment concept that may be considered (class IIb, LOE B) as an alternative DAPT strategy for ACS patients [14]. So far, no dedicated investigations focused on the optimal DAPT duration and different DAPT strategies including DAPT de-escalation in ACS patients after BVS implantation. Against this background, we analysed the safety and efficacy of a PFT-guided DAPT de-escalation strategy in ACS patients treated with BVS implantation that were enrolled in the TROPICAL-ACS study.

## Materials and methods

### Study design and patients

TROPICAL-ACS was an investigator-initiated, randomized, parallel-group, open-label, assessor-blinded, multicentre trial in ACS patients undergoing PCI. The trial was overseen by an independent Data Safety Monitoring Board and monitored by an external research organization (Münchner Studienzentrum, Munich, Germany). The study was conducted in accordance to the Declaration of Helsinki and it was approved by the institutional ethics committee of each participating site, as well as by the associated competent national agencies. Biomarker positive ACS patients aged ≥ 18 and ≤ 80 years were enrolled after successful PCI with a metallic st ent or a bioresorbable scaffold implantation determined by operator discretion. Key exclusion criteria were a history of TIA or stroke and need for oral anticoagulation. More details on inclusion and exclusion criteria were published previously [1]. In this specific post-hoc analysis we investigated the study subgroup of patients who received an ABSORB (Abbott) bioresorbable vascular scaffold (BVS) during the index PCI procedure.

### Randomization and study groups

ACS patients in this trial were randomized prior to discharge in a 1:1 fashion to groups of either (I) DAPT de-escalation guided by platelet function testing (PFT) or (II) a uniform treatment with prasugrel (control group). During the study period of 12 months, control group patients received prasugrel treatment (5 or 10 mg/day) according to current guideline recommendations [13]. In the guided de-escalation group, patients received a post-discharge treatment, consisting of 1-week prasugrel treatment followed by 1-week clopidogrel treatment (75 mg/days). PFT on clopidogrel was performed 2 weeks after hospital discharge. Based on PFT results in the guided de-escalation group, patients were either switched back to prasugrel, when a status of high platelet reactivity (HPR, for definition see below) was detected, whereas patients with sufficient platelet inhibition (no HPR) continued with clopidogrel.

### Follow-up procedures and PF testing

Two weeks after discharge all patients had an outpatient visit that included blood sampling for PFT on the Multiplate analyzer (Roche Diagnostics, Rotkreuz, Switzerland). Details of this whole-blood based method have been published previously and a status of HPR for this assay was defined according to the current consensus document of the Working Group on HPR by an ADPtest aggregation value of ≥ 46 U [1, 15]. In the guided de-escalation group, testing results determined the further course of treatment. For observational purposes and to achieve equal conditions in both study groups control group patients were also seen in hospital for this follow-up with PFT. For the clinical follow-up, patients were contacted by a phone call at 30 days, 6 and 12 months after randomization.

### Study endpoints

The primary endpoint was a net clinical benefit endpoint defined as combined ischemic and bleeding events, consisting of death from cardiovascular cause (CVD), myocardial infarction (defined according to the 3rd universal definition of MI), nonfatal stroke and bleeding grade ≥ 2 defined according to BARC criteria at 1-year after randomization [9, 10]. The key secondary endpoint was defined as class ≥ 2 bleeding events at 12 months defined according to BARC criteria. Further details on study endpoints were reported previously [1].

### Statistical analysis

As this was a post-hoc subgroup analysis from a randomized controlled clinical trial, no specific statistical assumptions for the sample size were made beforehand. All analyses on outcomes in BVS patients were done on an intention-to-treat basis. The hazard ratios (HR) for different outcomes were evaluated in univariate Cox-regression models according to randomized groups and Kaplan–Meier plots were generated to visualize the risk of outcome events in both groups. Binary and other categorical variables were compared using Fisher’s exact test and χ^2^ test, respectively, for continuous data two-sided unpaired Wilcoxon test or Student’s *t-*test were used as appropriate. Data were analysed with R version 3.3.0.

## Results

**Fig. 1:**
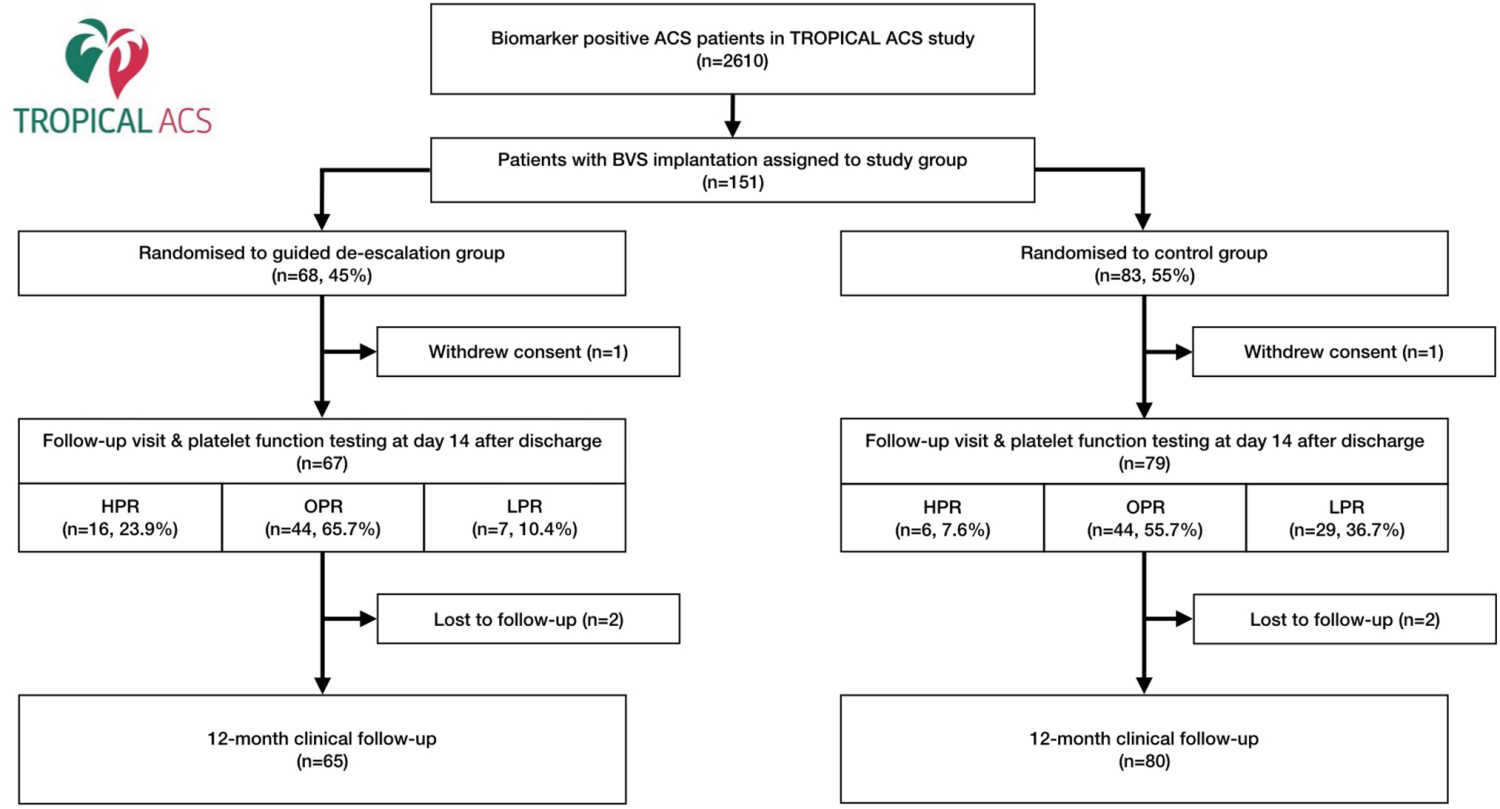
Study flow chart. *ACS* acute coronary syndrome, *BVS* bioresorbable vascular scaffold, *HPR* high platelet reactivity, *OPR* optimum platelet reactivity, *LPR* low platelet reactivity

From the entire TROPICAL-ACS study (n = 2,610) 151 patients (5.8%) received a BVS during their index ACS-PCI procedure. Eighty-three patients were part of the control group and 68 patients were part of the guided de-escalation group. A detailed description of the flow of patients for this specific analysis is presented in Fig. 1. Baseline and procedural characteristics are summarized in Tables 1 and 2.

**Table 1:** Baseline clinical characteristics

	Control group (n = 83)	Guided de-escalation group (n = 68)	P-value
Age (years)	57.8 (12.1)	56.2 (9.2)	0.35
Female	15 (18.1)	17 (25)	0.32
Body Mass Index (kg/m^2^)	27.7 (3.8)	28 (4.5)	0.61
Caucasian race	83 (100)	68 (100)	–
Previous percutaneous coronary intervention	7 (8.4)	4 (5.9)	0.75
Previous coronary artery bypass surgery	0 (0)	1 (1.5)	0.45
Previous myocardial infarction	7 (8.4)	5 (7.4)	> 0.99
History of peripheral artery occlusive disease	4 (4.8)	0 (0)	0.13
History of coronary artery disease	6 (7.2)	8 (11.8)	0.40
Renal insufficiency	2 (2.4)	6 (8.8)	0.14
Diabetes mellitus	11 (13.3)	11 (16.2)	0.65
Current smoker	35 (42.2)	38 (55.9)	0.24
Arterial hypertension	45 (54.2)	40 (58.8)	0.85
Hyperlipidaemia	30 (36.1)	21 (30.9)	0.89
Family history of coronary artery disease	22 (26.5)	22 (32.4)	0.47
LVEF (%)	54.6 (9.8)	56.3 (7.8)	0.33
Haemoglobin (g/dL)	14.3 (1.8)	14.3 (2.1)	0.82
Creatinine (mg/dL)	1.0 (0.2)	1.0 (0.4)	0.26
Medication at admission			
Aspirin	10 (12)	11 (16.2)	0.49
ADP receptor antagonist	4 (4.8)	2 (2.9)	0.69
Beta blocker	15 (18.1)	17 (25)	0.32
ACE inhibitor	14 (16.9)	13 (19.1)	0.83
Angiotensin1 receptor antagonist	12 (14.5)	12 (17.6)	0.66
Calcium antagonist	5 (6.0)	10 (14.7)	0.10
Proton-pump inhibitor	6 (7.2)	6 (8.8)	0.77
Statin treatment	13 (15.7)	15 (22.1)	0.40

Data are n (%) or mean (SD), *LVEF* left ventricular ejection fraction (%), *ADP* adenosine diphosphate, *ACE* angiotensin-converting-enzyme

**Table 2:** Angiographic and procedural characteristics

	Control group (n = 83)	Guided de-escalation group (n = 68)	P-value
Cause of PCI			
STEMI	43 (51.8)	33 (48.5)	0.74
NSTEMI	40 (48.2)	35 (51.5)	
Access site			
Femoral	42 (50.6)	36 (52.9)	0.96
Radial	41 (49.4)	32 (47.1)	
Number of diseased coronary arteries			
1	46 (55.4)	36 (52.9)	0.95
2	21 (25.3)	19 (27.9)	0.94
3	16 (19.3)	13 (19.1)	> 0.99
Anticoagulant agent used for PCI			
Bivalirudin	1 (1.2)	2 (2.9)	0.75
Low molecular weight heparin	1 (1.2)	0 (0)	0.66
Unfractionated heparin	81 (97.6)	66 (97.1)	0.98
Use of glycoprotein IIb/IIIa antagonist	11 (13.3)	10 (14.7)	0.82
TIMI flow grade before PCI			
0	33 (39.8)	22 (32.4)	0.64
1	7 (8.4)	7 (10.3)	0.93
2	21 (25.3)	17 (25)	> 0.99
3	22 (26.5)	22 (32.4)	0.73
Coronary vessels treated			
Left main	0 (0)	0 (0)	–
Left anterior descending	37 (44.6)	30 (44.1)	> 0.99
Left circumflex	14 (16.9)	17 (25)	0.47
Right coronary artery	30 (36.1)	21 (30.9)	0.79
Coronary bypass graft	2 (2.4)	0 (0)	0.44
AHA/ACC classification of lesions			
A	17 (20.5)	11 (16.2)	0.80
B1	21 (25.3)	24 (35.3)	0.41
B2	20 (24.1)	19 (27.9)	0.87
C	25 (30.1)	14 (20.6)	0.41
Ostial lesion	5 (6.0)	2 (2.9)	0.46
Bifurcation lesion	2 (2.4)	2 (2.9)	> 0.99
TIMI flow grade after PCI			
2	7 (8.4)	2 (2.9)	0.19
3	76 (91.6)	66 (97.1)	

Data are n (%), *STEMI* ST-segment elevation myocardial infarction, *NSTEMI* non-ST-segment elevation myocardial infarction, *PCI* percutaneous coronary intervention, *TIMI* thrombolysis in myocardial infarction, *AHA* American Heart Association, *ACC* American College of Cardiology

**Fig. 2:**
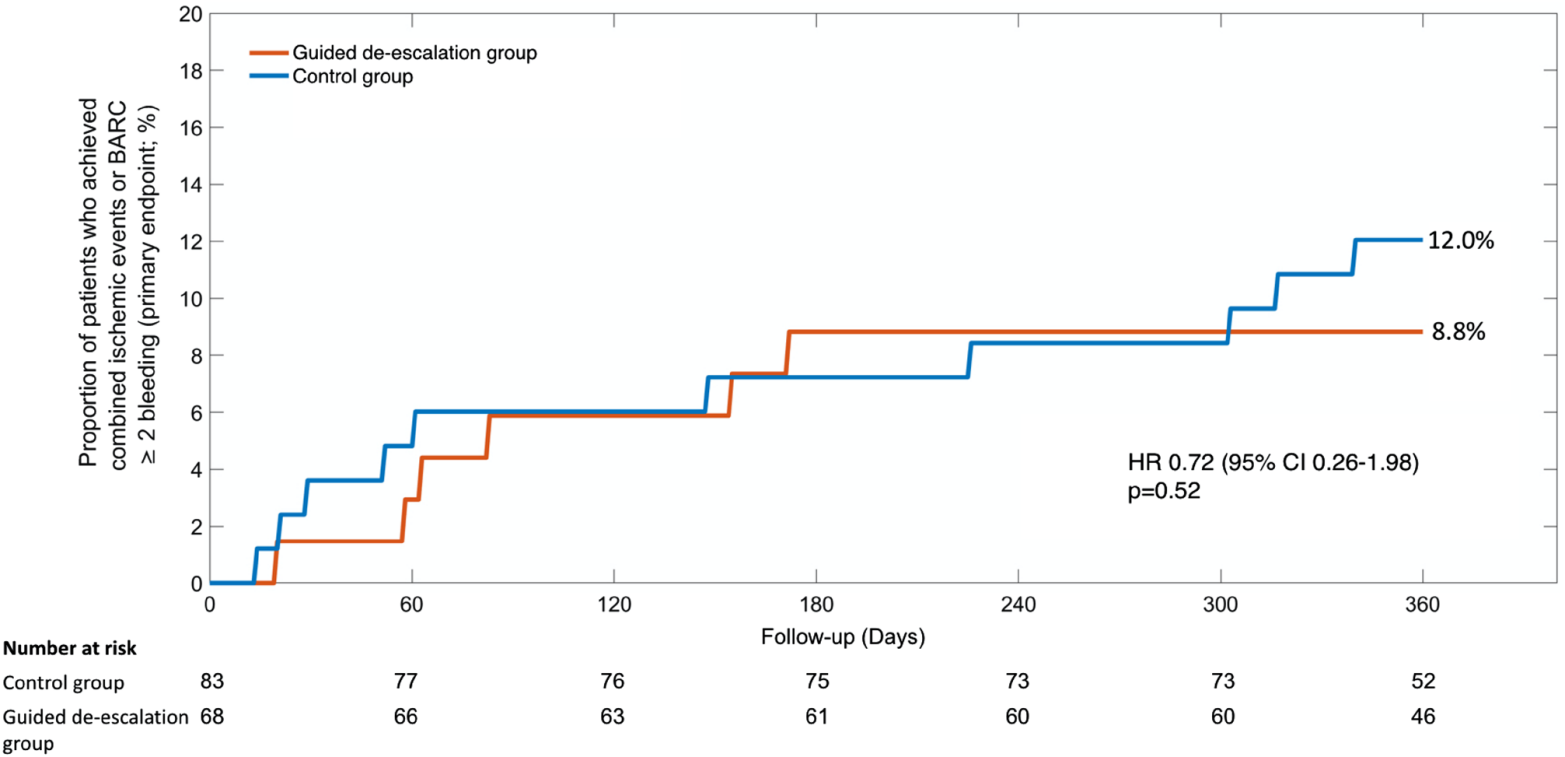
Kaplan–Meier curves for the primary endpoint (net clinical benefit). *BARC* Bleeding Academic Research Consortium, *HR* Hazard ratio, *95% CI* 95% confidence interval

The primary combined endpoint (cardiovascular death, myocardial infarction, stroke or bleeding ≥ grade 2 according to BARC criteria) occurred in 6 patients (8.8%) in the de-escalation group (n = 68) and in 10 patients (12.0%) in the control group (n = 83) (HR 0.72, 95% CI 0.26–1.98, p = 0.52) (Fig. 2). A Cox proportional hazards model that included presence (in n = 151 patients) vs. absence (in n = 2459 patients) of a BVS as a dichotomic variable showed no interaction of BVS implantation with treatment effects for the primary endpoint between study groups (p-value for interaction = 0.82).

The incidence of the key secondary endpoint of BARC ≥ 2 or higher bleedings was 5.9% (4 events) in the guided de escalation group versus 8.4% (7 events) in the control group [HR 0.69 (95% CI 0.20–2.67); p = 0.55]. The cumulative incidence of all bleeding events (BARC class 1 to 5) was 10.3% (7 events) in the guided de escalation group versus 9.6% (8 events) in the control group [HR 1.09 (95% CI 0.39–2.99); p = 0.87].

Combined ischemic events (cardiovascular death, myocardial infarction, and stroke) occurred in 2 patients (2.9%) in the guided de-escalation group and in 4 patients (4.8%) in the control group [HR 0.61 (95% CI 0.11–3.32); p = 0.56], suggesting that early de-escalation did not result in an increased ischemic risk. Further details on outcomes across study groups are reported in Table 3.

**Table 3:** Clinical outcomes at 12 months

	Control group (n = 83)	Guided de-escalation group (n = 68)	Hazard ratio [95% CI]	P-value
Net clinical benefit				
Primary endpoint (cardiovascular death, myocardial infarction, stroke, bleeding BARC ≥ 2)	10 (12)	6 (8.8)	0.72 [0.26–1.98]	0.52
Ischaemic events				
Combined ischaemic events (cardiovascular death, myocardial infarction, stroke)	4 (4.8)	2 (2.9)	0.61 [0.11–3.32]	0.56
Cardiovascular death	0 (0)	1 (1.5)	–	0.93
Myocardial infarction	4 (4.8)	0 (0)	–	0.86
Stroke	0 (0)	1 (1.5)	–	0.93
Stent thrombosis (definite)	1 (1.2)	0 (0)	–	0.93
All-cause mortality	0 (0)	1 (1.5)	–	0.93
Urgent revascularisation	3 (3.6)	1 (1.5)	0.40 [0.04–3.87]	0.43
Bleeding events				
Key secondary endpoint (BARC bleeding ≥ 2)	7 (8.4)	4 (5.9)	0.69 [0.20–2.36]	0.55
BARC type 1 or 2	8 (9.6)	6 (8.8)	0.93 [0.32–2.67]	0.89
BARC type 3 or 5	0 (0)	1 (1.5)	–	0.93
Any BARC bleeding	8 (9.6)	7 (10.3)	1.09 [0.39–2.99]	0.87

Data are n (%), P values presented are for superiority comparisons unless otherwise stated, *BARC* Bleeding Academic Research Consortium

The overall frequency of definite ST at 1 year in the TROPICAL-ACS subset of patients with BVS implantation was 0.7%. We observed only one early scaffold thrombosis (ST) event in the control group in a 57-year-old male patient that 18 days after hospital discharge presented with clinical symptoms of a ST segment elevation myocardial infarction. Despite being treated with 10 mg/d of prasugrel, the patient exhibited HPR (ADPtest of 108 U on prasugrel and assessed at day 14 after discharge). Per study protocol, antiplatelet treatment remained unchanged for this patient in the control group. Further on, there was one possible ST (sudden CVD) in the guided de-escalation group at day 86 after randomization. This was a 58-year-old male, who presented with a NSTEMI during the index ACS-PCI procedure. This patient exhibited HPR as well (ADPtest of 59 U on clopidogrel assessed at day 14 after discharge) and according to the study protocol the patient was switched to 10 mg/days prasugrel thereafter.

## Discussion

To the best of our knowledge this is the first study providing clinical outcomes on a DAPT de-escalation regimen in ACS patients with BVS implantation [15]. The key findings of our study are that (i) a guided DAPT de-escalation was found to be safe in BVS patients when compared to a standard regimen of uniform platelet inhibition and that (ii) overall ischemic event rates including ST were comparatively low while the few events that occurred were observed in patients with HPR at the time point of PFT assessment. In general, our findings are in line with the main outcomes of the randomized TROPICAL-ACS trial, in which a guided DAPT de-escalation strategy was shown to be non-inferior to standard and uniform treatment with prasugrel [2]. It must be acknowledged, however, that due to the overall small number of BVS treated patients in the TROPICAL-ACS trial, the present results, albeit important and novel, must be interpreted with caution and should be considered as hypothesis generating.

Current ESC guideline recommendations on the duration of DAPT after BVS implantation are based solely on expert opinions and advocate a prolonged platelet inhibition believed to reduce the rate of ST [13]. Such a general statement may unnecessarily increase patient´s bleeding risk, particularly when potent ADP receptor blockers are included in a DAPT regimen. Our data bring new light into the field, by indicating that an early and guided DAPT de-escalation, with a switch from prasugrel to clopidogrel, might be a safe alternative even for BVS treated patients. Present data from this post-hoc analysis gains in importance since the recent ESC guidelines have included a new recommendation for guided DAPT de-escalation [14]. At this time, it must be acknowledged that ischemic event rates were low in the subset of BVS patients and that such a post-hoc analysis in a small cohort of patients is by definition not powered to provide definite answers in this respect. However, in light of the absence of DAPT de-escalation data in BVS patients, our results are novel and should build a basis for studying this and other alternative DAPT regimens in those patients.

Interestingly, despite a guided DAPT de-escalation, the rates of scaffold thrombosis in our analysis were lower than those previously reported for BVS in the literature (15–17). The latest meta-analyses comparing BVS to DES showed a twofold increase in definite/probable device thrombosis at 12 months (1.6% vs. 0.6%), and a threefold increase at 2 years after BVS implantation (2.3% vs. 0.7%) [10, 16]. In our BVS study cohort we found a comparably low incidence (0.7%) of definite or probable scaffold thrombosis, which may at least in part result from a closer DAPT monitoring resulting in a very high adherence to antiplatelet treatment during the first year after PCI [2]. Interestingly, the few ST cases (1 definite ST, 1 possible ST) that occurred had a documented status of HPR at 14 days after hospital discharge. While a play of chance cannot be excluded in such low event numbers, these observations suggest that the level of platelet reactivity may be linked to outcomes in BVS patients. This finding is in line with the results of a large collaborative meta-analysis in > 20.000 patients, where HPR was associated with ST risk and this association was valid for both prasugrel and clopidogrel treated patients [17].

The promise of clinical advantage of BVS over current metallic stent technology includes a reduction in long-term adverse events stemming from permanent stent, feasibility of non-invasive imaging and maintaining suitability for future treatment options including CABG. Enthusiasm for the encouraging early clinical outcomes of BVS, which showed acute performance comparable to DES, has been mitigated by the mid- and long-term increased incidence of adverse events, which revealed a twofold higher rate of stent thrombosis and target lesion failure [9, 18]. However, most of the clinical evidence on safety and efficacy of scaffolds came from the first generation Absorb BVS (Abbott, US) [9]. This device is characterized by thick (~ 150 µm) and wide (~ 200 µm) struts, altered shear stress that may activate platelets and a delayed resorption process resulting in intraluminal scaffold dismantling [19]. Currently, the second-generation BVS are being introduced with markedly thinner struts (~ 100 µm), improved expansion, self-correcting features and a more rapid resorption process [20]. Presently, there are over 22 s-generation BVS in different stages of development, of which five are CE-marked and commercially available in Europe. One year clinical data is very promising by showing similar or even lower event rates of ST in second generation BVS 0.0% MAGMARIS [Biotronik], 0.4% FANTOM [Reva Medical], 0.8% (DESOLVE [Elixir Medical]) compared to the first generation (0.8% ABSORB [Abbott]) [7, 21–23]. During long-term follow-up, the second generation BVS demonstrated numerically lower rates of ST (0.0% at 24 months for MAGMARIS [Biotronik] and 0.0% from years 2 through 5 for DESOLVE [Elixir Medical]), compared to the first generation (3% at 36 months for ABSORB [Abbott]) [7, 24]. With respect to the analysis presented here, the observed safety and efficacy of an early PFT-guided DAPT de-escalation strategy in the first generation BVS could potentially be extrapolated to BVS patients in the future, who may receive a 2nd generation BVS. Furthermore, the new BVS are intended for simple lesions commonly seen in younger patients and this subset of patients achieved a net clinical benefit during a guided DAPT de-escalation that was mainly driven by a reduction in bleeding events [3].

## Limitations

There are number of limitations that merit being mentioned. First, due to the overall small number of BVS-treated patients in the TROPICAL-ACS trial, our post-hoc analysis was not powered for clinical endpoints and the presented results must be interpreted with caution and should be considered as hypothesis generating. However, with 151 BVS patients included, this is the largest subset of patients from a randomized clinical trial which evaluated the safety and efficacy of a DAPT de-escalation strategy. Second, a limited cohort size precluded stratifying the population into further subgroups according to platelet response phenotype (HPR or LPR), though all the ScT cases occurred in patient with HPR. Third, all patients in our cohort received a first generation BVS, which has been withdrawn from the market by the producer. However, we could assume that as the de-escalation strategy could also work in the new 2nd generation BVS platforms that are characterized by thinner struts, this however requires future research. Finally, the TROPICAL-ACS trial was designed only with a 12 months follow-up period and is consistent with the current DAPT de-escalation strategies that follow the approach of an early switch from potent agents over to clopidogrel [2, 25]. Thus a DAPT de-escalation strategies that cover the treatment beyond 1 year after ACS require separate investigations.

## Conclusions

A PFT-guided DAPT de-escalation strategy could potentially be a safe and effective strategy in ACS patients with BVS implantation. The level of platelet inhibition may be of particular importance in this subset of patients and a DAPT monitoring by PFT may be a useful tool to achieve sufficient platelet inhibition and to increase overall DAPT adherence. Although this is a post-hoc sub-analysis of BVS patients, it carries a considerable hypothesis hypothesis-generating value needed to design larger studies with next generation BVS platforms.
